# Evaluation of Leading Smartwatches for the Detection of Hypoxemia: Comparison to Reference Oximeter

**DOI:** 10.3390/s23229164

**Published:** 2023-11-14

**Authors:** Simon Walzel, Radek Mikus, Veronika Rafl-Huttova, Martin Rozanek, Thomas E. Bachman, Jakub Rafl

**Affiliations:** Department of Biomedical Technology, Faculty of Biomedical Engineering, Czech Technical University in Prague, 272 01 Kladno, Czech Republichuttover@fbmi.cvut.cz (V.R.-H.); rozanek@fbmi.cvut.cz (M.R.); tbachman@me.com (T.E.B.); rafl@fbmi.cvut.cz (J.R.)

**Keywords:** smartwatch, wearables, oxygen saturation, hypoxemia, pulse oximetry, reflectance mode, hypoxic gas mixture

## Abstract

Although smartwatches are not considered medical devices, experimental validation of their accuracy in detecting hypoxemia is necessary due to their potential use in monitoring conditions manifested by a prolonged decrease in peripheral blood oxygen saturation (SpO_2_), such as chronic obstructive pulmonary disease, sleep apnea syndrome, and COVID-19, or at high altitudes, e.g., during sport climbing, where the use of finger-sensor-based pulse oximeters may be limited. The aim of this study was to experimentally compare the accuracy of SpO_2_ measurement of popular smartwatches with a clinically used pulse oximeter according to the requirements of ISO 80601-2-61. Each of the 18 young and healthy participants underwent the experimental assessment three times in randomized order—wearing Apple Watch 8, Samsung Galaxy Watch 5, or Withings ScanWatch—resulting in 54 individual experimental assessments and complete datasets. The accuracy of the SpO_2_ measurements was compared to that of the Radical-7 (Masimo Corporation, Irvine, CA, USA) during short-term hypoxemia induced by consecutive inhalation of three prepared gas mixtures with reduced oxygen concentrations (14%, 12%, and 10%). All three smartwatch models met the maximum acceptable root-mean-square deviation (≤4%) from the reference measurement at both normal oxygen levels and induced desaturation with SpO_2_ less than 90%. Apple Watch 8 reached the highest reliability due to its lowest mean bias and root-mean-square deviation, highest Pearson correlation coefficient, and accuracy in detecting hypoxemia. Our findings support the use of smartwatches to reliably detect hypoxemia in situations where the use of standard finger pulse oximeters may be limited.

## 1. Introduction

Nowadays, smartwatches offer much more than simply connecting to a mobile phone and managing basic functions. Recent studies have shown that, thanks to integrated sensors, smartwatches can monitor heart rate, ECG, and blood pressure, or measure peripheral blood oxygen saturation (SpO_2_) values [1,2,3,4]. The advantage of real-time monitoring of vital signs using smartwatches is their convenient placement on the forearm, which does not restrict the user in daily activities compared to conventional blood pressure cuffs or finger sensors for SpO_2_ measurement. Furthermore, users can customize the device to their needs and view the recorded data, which can help in the diagnosis, prevention, and possibly management of various diseases [5]. Atrial fibrillation is an example of a life-threatening condition that smartwatches can diagnose very accurately [6]. With the onset of the global COVID-19 pandemic, smartwatch data have also been shown to identify those infected among symptomatic individuals [7]. Smartwatch-based SpO_2_ monitoring has been discussed primarily in patients suffering from chronic obstructive pulmonary disease (COPD) [8], sleep apnea syndrome [9], and COVID-19 disease [10,11]. These diseases are often characterized by a prolonged decrease in SpO_2_ levels where measurement with a standard fingertip oximeter is limiting for the patient. Physiological SpO_2_ values in a healthy individual are in the range of 95–100% but are typically reduced to 88–92% in an individual with acute or chronic cardiopulmonary problems [12]. Smartwatch-based SpO_2_ monitoring may also be used to predict acute mountain sickness [13].

Smartwatches, as well as, for instance, fitness trackers, belong to a group of electronic devices commonly referred to as ‘wearable devices’. The reflectance method used in wearable devices to measure SpO_2_ has the problems of a significantly lower signal-to-noise ratio compared to the standard transmission method used in clinical practice and the much smaller perfusion of the wrist compared to the finger [14,15]. Motion artifacts are an issue, being more common in wrist measurements than in fingers due to the presence of tendons and bones. If the sensor is not pressed firmly against the tissue, artifacts due to ambient lighting also occur [16]. In addition, a study by Apple [17] describes a possible problem with the deteriorated quality of the signal from the photoplethysmographic sensor in people with darker skin, which applies to all oximeters. However, it appears that despite the many complications of wrist-based measurements, it is possible to achieve high accuracy in determining the final SpO_2_ value through hardware and software optimization [14,15,18]. In addition, studies validating the SpO_2_ accuracy of wrist- or finger-worn wearables have demonstrated their ability to achieve clinically sufficient accuracy over the range of oxygen saturation values examined [19,20,21].

ISO 80601-2-61 is the international standard for assessing the accuracy of SpO_2_ measurements by pulse oximeters [22]. The use of this standard requires, among other things, the induction of short-term hypoxemia in participants and allows validation of the accuracy of SpO_2_ measurements in both invasive and non-invasive ways. The accuracy (determined as the root-mean-square difference, A_rms_) of the pulse oximetry device over the SpO_2_ range of 70% to 100% must be better than or equal to 4.0% compared to the reference device. The U.S. Food and Drug Administration (FDA) has established a stricter criterion for validating the accuracy of SpO_2_ measurements by pulse oximeters. For an oximeter with a reflectance method of measuring SpO_2_, a root-mean-square difference of better than or equal to 3.5% is required [23]. In a study by Kirszenblat and Edouard [16] that followed this standard, the SpO_2_ value determined by Withings ScanWatch was compared with the arterial oxygen saturation (SaO_2_) value measured by a blood gas analyzer. A study by Apple [17], which followed the development of an SpO_2_ measurement app used in their smartwatch, also adhered to the standard, using the invasive measurement as a reference. Both studies found minimal differences in SpO_2_ measured by the smartwatch compared to the gold standard. A non-invasive approach to validate the accuracy of SpO_2_ measurement using Apple Watch 6 compared to a standard oximeter was taken in our previous study [24]. The results of the study also demonstrated the high accuracy of the smartwatch in detecting hypoxemia. Lauterbach et al. [25] used a different approach to test the smartwatch using a normobaric hypoxic chamber but again found only minimal differences in SpO_2_ measurements when compared to a standard oximeter. The above studies [16,17,24,25] were concerned with determining the accuracy of smartwatch SpO_2_ measurements in a group of healthy volunteers during hypoxemia only. Several other studies have investigated the accuracy of Apple Watch SpO_2_ measurement in a group of patients with pathologically impaired SpO_2_ values [26,27,28,29]. The measurement methods and the conclusions of the studies on the clinical use of smartwatches vary, but the results show relatively little systematic bias between the devices tested. Recently, Schroder et al. [30] pointed out a potential problem with outliers in smartwatch SpO_2_ measurements, as some of the values measured by the watch compared to a standard oximeter lay outside the physiological range of 95–100%, even though the measurements were performed on healthy volunteers under normal conditions.

Only two studies [16,17] have validated the accuracy of SpO_2_ measurements even at SpO_2_ levels below 80% and fully complied with ISO 80601-2-61. While other manufacturers are introducing their smartwatches capable of measuring SpO_2_, according to the available literature, no study has been conducted that simultaneously compares multiple smartwatch models using a single measurement method while meeting the criteria set by the above-mentioned standard.

The aim of this study was to experimentally compare the accuracy of several smartwatches with a clinically used pulse oximeter in the SpO_2_ range of 70–100%.

## 2. Methods

This prospective, interventional, randomized crossover study was approved by the Ethical Review Board of the Faculty of Biomedical Engineering of the Czech Technical University in Prague on 7 February 2023 (no. C27/2023). All participants provided written consent prior to enrollment. The study has been registered with ClinicalTrials.gov (NCT05789563).

A total of 18 healthy Caucasian volunteers (14 males, 4 females) aged 21–26 years participated in the study; the group characteristics are shown in Table 1. The number of participants enrolled in the study and the number of paired SpO_2_ observations were based on the International Organization for Standardization (ISO 80601-2-61:2019) guideline for in vivo accuracy testing of pulse oximeters, which requires at least 200 paired SpO_2_ readings balanced across the SpO_2_ range of 70–100% from at least 10 subjects [22]. Screened before enrollment, no participants were excluded from the study because of cardiovascular or respiratory disease, pregnancy, diabetes, any acute illness, or upper limb or hand injury that could affect peripheral perfusion.

Each participant underwent the experimental assessment three times in a randomized order, wearing one of three smartwatches (Apple Watch 8 (Apple Inc., Cupertino, CA, USA), Samsung Galaxy Watch 5 (Samsung Electronics Co., Ltd., Suwon-si, Republic of Korea), or Withings ScanWatch (Withings, Issy-les-Moulineaux, France)). The order in which the smartwatches were worn was assigned using computer-generated random numbers. At least a 2 h recovery interval was included between the experimental assessments.

Upon arrival at the workplace, the participants were seated in a comfortable position with their left hand placed on the table in front of them near heart level and with the wrist and palm facing down. A smartwatch (hereafter referred to as Apple, Samsung, or Withings) was attached to the participant’s left wrist according to the manufacturer’s instructions. The Radical-7 reference pulse oximeter sensor (Masimo Corporation, Irvine, CA, USA) was placed on the left middle finger. Three test SpO_2_ readings from the smartwatch were always taken before the start of the experimental assessment. If the three consecutive readings did not indicate SpO_2_ greater than 90%, the position of the smartwatch was adjusted, and the test readings were repeated.

A non-rebreathing circuit was set up for the experimental assessments. It allowed the participant to inhale either a hypoxic gas mixture from the Douglas bag or the ambient air and exhale into the ambient air outside the Douglas bag. Inhalation was performed through an anesthetic mask covering the mouth and nose. The composition of the inhaled gas mixtures was monitored continuously by a Datex-Ohmeda S/5 patient monitor (Datex-Ohmeda Inc., Madison, WI, USA) with a sensor placed in the breathing circuit. A disposable antibacterial filter separated the participant from the breathing circuit.

There were three phases in each of the 12 min experimental assessments. During the first 2 min, in the initial stabilization phase, participants inhaled the ambient air via the non-rebreathing circuit. This was followed by the 7.5 min desaturation phase, during which participants inhaled the hypoxic gas mixture from the Douglas bag. Three different hypoxic gas mixtures (14% O_2_, 12% O_2_, 10% O_2_) were used consecutively under normobaric conditions during the desaturation phase (2.5 min each), which we expected to cover the desired saturation range. The reduced oxygen content corresponds approximately to altitudes of 3200 m (14% O_2_), 4400 m (12% O_2_), and 5800 m (10% O_2_). The final stabilization phase, during which participants inhaled ambient air through the breathing circuit, lasted until stable readouts were reached.

Manual readings of SpO_2_ values from the smartwatch and the reference oximeter were taken simultaneously at predefined time points during the experimental assessment at intervals of 40–50 s. The SpO_2_ value from the reference oximeter was obtained at the time the SpO_2_ reading from the smartwatch was completed. A total of 16 paired SpO_2_ readings were obtained from each experimental assessment.

### Data Processing

Only successful coupled readings from both the smartwatch and reference oximeter were included in the final analysis. All data were analyzed in Matlab 2021a (MathWorks, Natick, MA, USA) after transcription from the participant’s log.

To compare the three smartwatch models, each set of paired data was fitted with a linear regression line using the method of least squares, and the correlation coefficient was determined.

To assess the relative response of the smartwatch and the oximeter, we compared the SpO_2_ readings of all participants for both devices at each time point of the experimental assessment using a two-tailed paired *t*-test, with a *p*-value of less than 0.05 considered statistically significant.

The Bland–Altman analysis was conducted to evaluate the agreement between the SpO_2_ readings obtained from the smartwatch and the oximeter. This standard approach determines the scatter and bias between measurement methods. The 95% limits of agreement (LOAs) were calculated by adding and subtracting 1.96 standard deviations from the mean bias to provide an estimate of the expected differences between the simultaneous SpO_2_ readings acquired from the smartwatch and the oximeter. The standard deviation was calculated using the modified Bland–Altman method for multiple observations per individual when the measured quantity changes over the observation period. The mean bias was calculated as the average difference between the smartwatch and the oximeter measurements. Mean bias, LOAs, and A_rms_ between the smartwatch and the reference oximeter were also calculated for subintervals of the entire measured SpO_2_ range (100–91%, 90–81%, and ≤80%). Paired SpO_2_ readings were assigned to each interval according to the SpO_2_ value from the reference oximeter.

Finally, we evaluated the diagnostic sensitivity, specificity, and accuracy of each smartwatch in detecting hypoxemia, defined as SpO_2_ below 90% based on the reference oximeter, similar to the study by Santos et al. [19]. SpO_2_ values below 90% can be considered as a serious deterioration in oxygenation [31].

## 3. Results

The study was conducted on healthy volunteers at the Faculty of Biomedical Engineering in Kladno, Czech Republic, in the Laboratory of Special Equipment for ICU during March and April 2023. All 18 participants completed all three stages of the experimental assessment, resulting in 54 complete datasets with 864 paired manual SpO_2_ readings (288 for each smartwatch). Of the 864 total readings, 274 (95%) were successfully displayed for Apple, 283 (98%) for Samsung, and 238 (83%) for Withings, and, of the 795 total successful paired manual SpO_2_ readings, 454 (57%) were in the 91–100% SpO_2_ range, 229 (29%) were in the 81–90% SpO_2_ range, and 112 (14%) were in the sub-80% SpO_2_ range.

**Figure 1:**
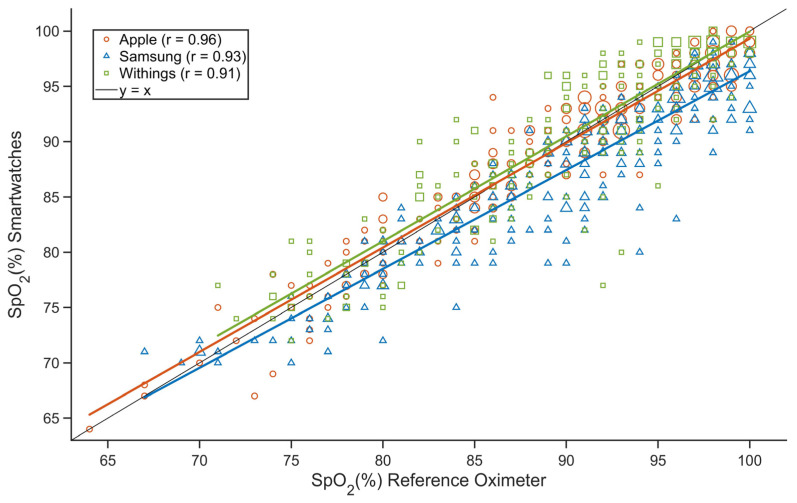
The relationship between the SpO_2_ value determined by the smartwatch and the SpO_2_ value determined by the reference oximeter (Apple—red, Samsung—blue, Withings—green). Pearson’s correlation coefficient is r. The markers in the graph are sized according to the frequency of observation of a given paired SpO_2_ reading from the smartwatch and the reference oximeter.

The individual datasets for all smartwatches were fitted with a regression line using the least squares method (Figure 1). While the regression line for Apple and Withings followed the ideal identity line well throughout the 70–100% SpO_2_ range, the difference between the SpO_2_ measured by Samsung and that measured by the reference oximeter decreased as SpO_2_ decreased. Pearson correlation coefficients were greater than 0.9 for all three smartwatches.

**Figure 2:**
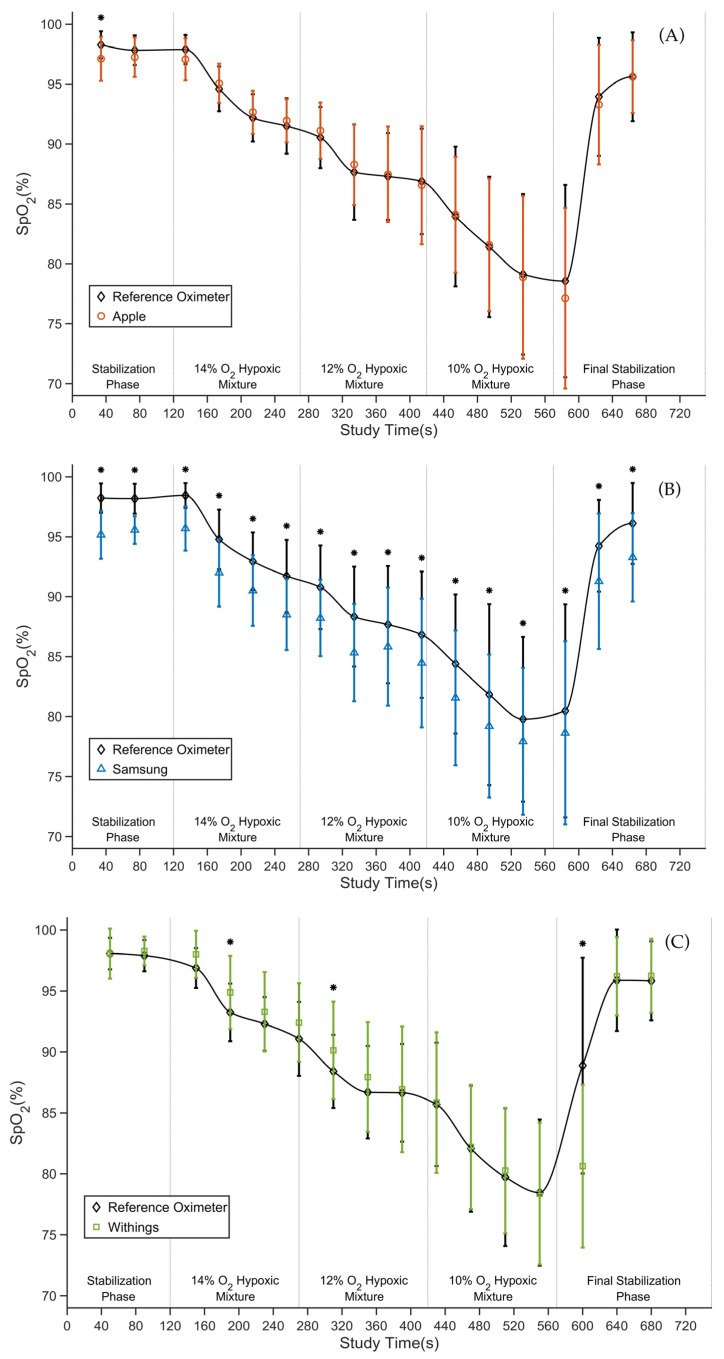
The time courses of the average SpO_2_ values of Apple Watch 8 (**A**), Samsung Galaxy Watch 5 (**B**), and Withings ScanWatch (**C**) compared to the Radical-7 reference pulse oximeter. Data are presented as mean ± standard deviation. * indicates a statistically significant difference.

The average SpO_2_ values measured by the smartwatch and the reference oximeter at each time point of the experimental assessment are depicted for each smartwatch in Figure 2A–C. The average SpO_2_ values measured by the reference oximeter for all experimental assessment stages decreased from 98% in the stabilization phase to about 78% at the end of the desaturation phase. The average difference between the smartwatch and the reference oximeter ranged from 0.0% SpO_2_ to −1.4% SpO_2_ for Apple, from −1.8% SpO_2_ to −3.2% SpO_2_ for Samsung, and from 0.0% SpO_2_ to −8.3% SpO_2_ for Withings. For Apple, there was only one statistically significant difference in the first manual reading (Figure 2A). For Samsung, there were statistically significant differences throughout the experimental assessment (Figure 2B), and for Withings, there were three statistically significant differences (Figure 2C).

**Figure 3:**
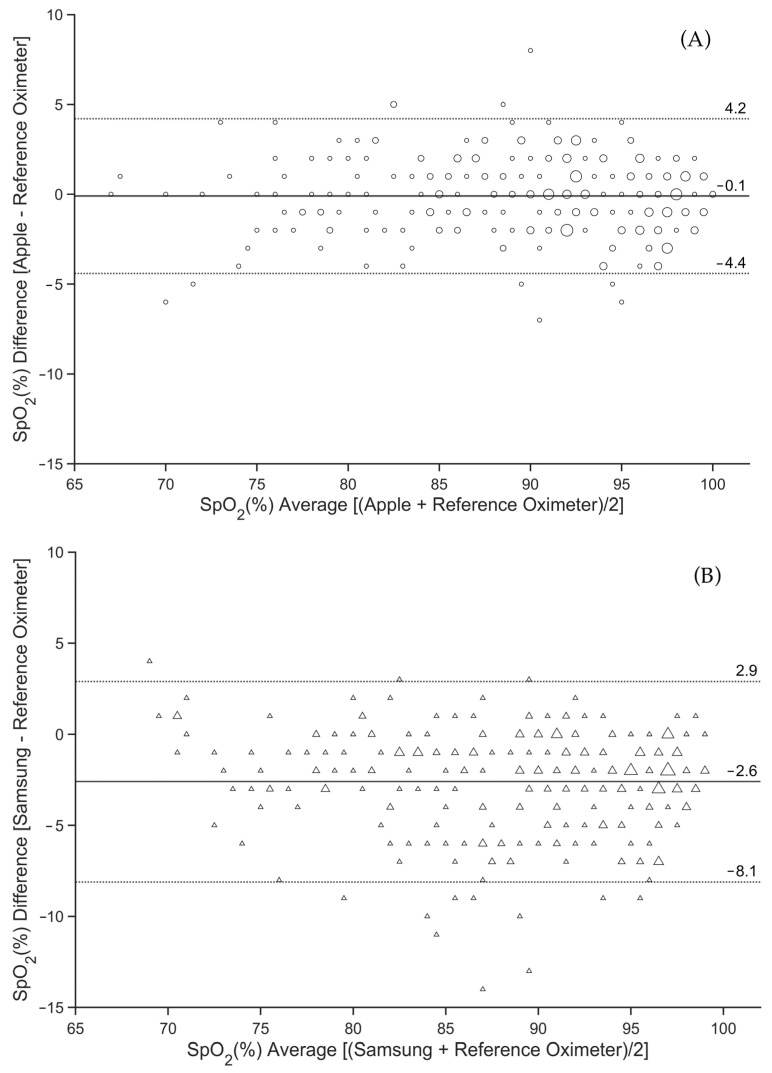

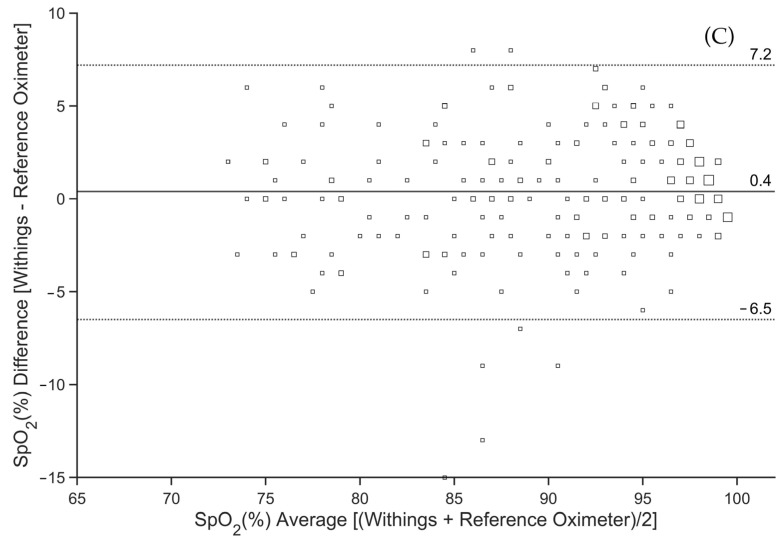
Differences between simultaneous SpO_2_ readings from Apple Watch 8 (**A**), Samsung Galaxy Watch 5 (**B**), and Withings ScanWatch (**C**) and from the Radical-7 reference pulse oximeter. The solid line is the mean bias of the readings. The dashed lines are the 95% limits of agreement. The markers in the graph are sized according to the frequency of observation of a given paired SpO_2_ reading from the smartwatch and the reference oximeter.

Bland–Altman plots that evaluate potential bias and limits of agreement between the smartwatch and the reference oximeter, derived from all pairs of pooled successfully obtained SpO_2_ readings, are displayed in Figure 3A–C. The mean bias in SpO_2_ values measured by the reference oximeter and Apple was −0.1% (Figure 3A), by the reference oximeter and Samsung was −2.6% (Figure 3B), and by the reference oximeter and Withings was 0.4% (Figure 3C). The 95% limits of agreement (LOAs) were found to be between −4.4% and 4.2% SpO_2_ for Apple, with the largest difference between the smartwatch SpO_2_ reading and the reference oximeter being −7% in the negative direction and 8% in the positive direction (Figure 3A). The 95% LOA for Samsung ranged from −8.1% to 2.9% SpO_2_, and the largest difference was −14% in the negative direction and 4% in the positive direction (Figure 3B). The 95% LOA for Withings ranged from −6.5% to 7.2% SpO_2_, and the largest difference was −15% in the negative direction and 8% in the positive direction (Figure 3C).

The comparison between the smartwatch and the reference oximeter was further analyzed after splitting into three SpO_2_ intervals (100–91%, 90–81%, ≤80%). The mean bias, lower and upper LOA, and A_rms_ are summarized in Table 2. As shown, Apple and Withings had mean bias not statistically different from zero in all intervals, except for the 90–81% SpO_2_ interval for Withings. In contrast, Samsung had a mean bias that was always statistically significant, although small. The widest range between lower and upper LOA was found for Withings and the narrowest for Apple. The calculated A_rms_ was less than 4% for all the smartwatches.

The reliability of smartwatches in detecting hypoxemia (SpO_2_ < 90%) is shown in Table 3. The negative mean bias in Samsung resulted in the highest sensitivity but the lowest specificity. The accuracy of Apple was statistically significantly higher than of the Samsung.

## 4. Discussion

In this experimental study, we directly compared the SpO_2_ measurements of three smartwatches from different manufacturers in young and healthy participants. Our main finding is that, although there are differences in the accuracy of SpO_2_ measurements between the smartwatches, these differences are small and of little importance to the average user. All three smartwatch models (Apple Watch 8, Samsung Galaxy Watch 5, and Withings ScanWatch) meet the accuracy requirements according to ISO 80601-2-61 when compared to the reference medical-grade pulse oximeter. However, in our study, only Apple Watch 8 and Withings ScanWatch met the more stringent FDA accuracy requirements.

When comparing the averages of paired readings between the smartwatch and the reference oximeter over time (Figure 2), a similar pattern of SpO_2_ decrease during the experimental assessment can be observed. Nevertheless, the only statistically significant difference for Apple was observed at the first time point of the SpO_2_ readings (Figure 2A), whereas, for Samsung, there was a statistically significant negative bias at all 16 time points of the SpO_2_ readings (Figure 2B). For Withings, the most significant differences occurred at 190 s, 310 s, and 600 s (Figure 2C). These differences may have been caused by the long interval required for the Withings smartwatch to determine the SpO_2_ value (30 s) compared to the 2–4 s averaging time of the reference oximeter. The longer time of SpO_2_ calculation may result in a deviation from the reference pulse oximeter when there is a rapid change in SpO_2_. Possible differences in calibration curves could also contribute to local differences.

The overall root-mean-square deviation of Apple (2.2%) is comparable to the value found in previous studies [17,24]. Samsung and Withings had A_rms_ values that were higher but still within the limits of the acceptable accuracy as specified by the ISO standard. Apple and Withings also showed a mean bias of less than 1% (Figure 3), which is completely negligible from a clinical point of view. In studies by Apple [17], Rafl et al. [24], and Pipek et al. [27], a mean bias of less than 1% in SpO_2_ was also observed for Apple smartwatches. Samsung had the largest mean bias of −2.6%. For Withings and Samsung, there was a decrease in A_rms_ and the mean bias at lower SpO_2_ values. However, this trend is opposite for the Apple smartwatch and opposite to the findings of Kirszenblat and Edouard [16] for Withings.

The authors of this study believe that the three selected smartwatch models appropriately represent the global market, as Apple’s watch market share reached 43% in terms of shipments in Q4 2022, making it the leading vendor. Samsung was next in line with 8% followed by Huawei, Amazfit, Garmin, Withings, and others. This distribution has not changed significantly over the years [32]. It is interesting to note that Withings ScanWatch is the only smartwatch on the market with FDA clearance for the functions of monitoring abnormal heart rhythms using ECG and alerting for breathing problems during the night using SpO_2_ measurement [33].

For Apple, of the 288 paired SpO_2_ readings, 274 (95.1%) were successful overall, indicating the smartwatch was properly attached to the wrist. The success rate for SpO_2_ readings is comparable to the results of the Apple study [17], which was 94.7%. Samsung achieved an even higher success rate for paired SpO_2_ readings in this study (98.3%). In contrast, Withings achieved a success rate of only 82.6%. In the study by Kirszenblat and Edouard [16], which also tested Withings ScanWatch, the success rate was comparable. Thus, it should be considered that, even under ideal measurement conditions (participants at rest with no movement and with the hand in front according to the manufacturer’s recommendations), there may be a number of failed SpO_2_ readings with some smartwatches.

All three smartwatch models demonstrated high diagnostic accuracy for hypoxemia (SpO_2_ <90%). Although the sensitivity was highest for Samsung (0.97), the smartwatch underestimated the SpO_2_ value compared to the reference oximeter throughout the measurement range and consequently had the lowest specificity value (0.76). However, the authors of this study suggest that this feature is preferable to overestimating SpO_2_ values.

This study had several limitations. First, only healthy Caucasian volunteers aged 21–26 years participated in the study. The gender imbalance of the study participants, which approximates the gender distribution of our students, may also be perceived as a limitation, but we did not expect this to significantly affect the results. Results may vary in chronic elderly patients or due to differences in skin pigmentation, which affects light transmission and reflectance. Second, the method of inducing hypoxemia did not allow stable SpO_2_ values or the same level of desaturation to be achieved in all participants. This also resulted in a relatively low number of SpO_2_ measurements below 80%. On the other hand, based on our experience with these types of hypoxic experiments, we believe that the method is a good compromise between slow desaturation, the reaching of relatively low stable SpO_2_ values, and the tolerable length of the experiment for the participants. Third, the SpO_2_ measurements were performed under laboratory conditions when the participants were at rest, comfortably seated, and the correct position of the smartwatch was verified. Thus, the success rate of SpO_2_ readings would likely be lower in routine practice, where the position of the smartwatch is not checked multiple times and the patient is not sitting perfectly still with their hand on the table in front of them. However, verifying the effects of the smartwatch position and motion artifacts on reading success rate was not the focus of this study. Next, we did not evaluate SaO_2_ in this study as it is a method that requires invasive arterial blood sampling, which greatly complicates the experimental assessment and increases the safety requirements for the participants. Finally, we do not know exactly how each smartwatch’s algorithms work to determine the final displayed SpO_2_ value. The smartwatches differ in the interval required to determine the final SpO_2_ value. For Apple Watch 8, the interval is 15 s. Samsung Galaxy Watch 5 does not have a fixed interval for determining the resulting value; it ranges between 12 and 17 s. Withings ScanWatch determines the SpO_2_ value in an interval of 30 s. This resulted in our inability to fully distinguish SpO_2_ measurement variations between devices from the time shift. For Apple and Samsung, the time shift was not apparent; however, for Withings, it appears that the difference between the reference oximeter and the smartwatch in the first half of the experimental assessment was due to the long interval required to determine the final SpO_2_ value, whereas, toward the end of the desaturation phase, this effect was no longer apparent (Figure 2C).

This study compared smartwatches from two top-selling smartwatch manufacturers as well as a smartwatch with FDA approval for detecting nighttime breathing problems. To our knowledge, no study has been conducted to compare multiple smartwatch models simultaneously using a single testing method. Therefore, we suggest the findings of the study can be applied to smartwatches in general with greater confidence than studies that validate a single model of a single manufacturer. Overall, the analysis of SpO_2_ measurements by smartwatches showed the high accuracy of these devices compared to a standard pulse oximeter. The differences we found are unimportant and likely to diminish as manufacturers introduce new models. Smartwatches are not intended for clinical SpO_2_ measurement, as the manufacturers themselves emphasize. Although the overall accuracy of smartwatches is sufficient, the long time needed to determine the SpO_2_ value and the high sensitivity to motion artifacts limit their potential clinical use. On the other hand, smartwatches allow long-term and continuous monitoring of SpO_2_ trends, detection of abnormal fluctuations, and, thus, faster evaluation of changes in the user’s health status over time. This is particularly advantageous for some groups of individuals, such as those suffering from chronic pulmonary disease, sleep apnea, or post-COVID syndrome.

## 5. Conclusions

Smartwatches from leading manufacturers do not show substantial differences from each other in SpO_2_ monitoring. They meet the accuracy requirement compared to the reference measurement both at normal oxygen levels and during induced desaturation with SpO_2_ below 90% in young and healthy people. Our findings support the general use of smartwatches to reliably detect hypoxemia in situations where the use of standard finger-sensor-based pulse oximeters may be limited.

## Figures and Tables

**Table 1:** The basic characteristics of the group of participants involved in the study.

Parameter	Participants (n = 18)
Age (years)	23.2 ± 1.8 (21–26)
BMI (kg·m^−2^)	24.6 ± 3.2 (19–30)
Systolic pressure (mmHg)	129 ± 7 (117–139)
Diastolic pressure (mmHg)	78 ± 9 (60–94)
Heart rate (bpm)	76 ± 13 (50–104)
Wrist circumference (cm)	18 ± 1.9 (15–23)

The values are presented as mean ± standard deviation (minimum–maximum). Abbreviation: BMI—Body Mass Index.

**Table 2:** Comparison of mean bias, lower and upper LOA, and root-mean-square deviation for SpO_2_ reading ranges 100–91%, 90–81%, and ≤80% between the smartwatch and the reference pulse oximeter.

Smartwatch	Measured SpO_2_ by Reference Oximeter, %	Mean Bias ^A^ (95% CI), %	Lower LOA (95% CI), %	Upper LOA (95% CI), %	A_rms_, %
Apple Watch 8	Full range (n = 274)	−0.1 (−0.4 to 0.1)	−4.4 (−4.9 to −4.0)	4.2 (3.7 to 4.6)	2.2
	100 to 91 (n = 156)	−0.5 (−0.8 to −0.2)	−4.5 (−5.0 to −3.9)	3.5 (2.9 to 4.0)	2.1
	90 to 81 (n = 76)	0.5 (0.0 to 1.0)	−3.7 (−4.6 to −2.9)	4.8 (3.9 to 5.6)	2.2
	≤80 (n = 42)	0.2 (−0.6 to 1.0)	−4.8 (−6.2 to −3.5)	5.2 (3.8 to 6.5)	2.5
Samsung Galaxy Watch 5	Full range (n = 283)	−2.6 (−2.9 to −2.3)	−8.1 (−8.7 to −7.6)	2.9 (2.4 to 3.5)	3.8
	100 to 91 (n = 164)	−3.1 (−3.5 to −2.7)	−8.5 (−9.2 to −7.7)	2.3 (1.6 to 3.0)	4.1
	90 to 81 (n = 79)	−2.2 (−2.9 to −1.6)	−8.1 (−9.3 to −6.9)	3.6 (2.5 to 4.8)	3.7
	≤80 (n = 40)	−1.3 (−2.1 to −0.6)	−6.0 (−7.4 to −4.7)	3.4 (2.0 to 4.7)	2.7
Withings ScanWatch	Full range (n = 238)	0.4 (−0.1 to 0.8)	−6.5 (−7.2 to −5.7)	7.2 (6.5 to 8.0)	3.5
	100 to 91 (n = 134)	−0.1 (−0.7 to 0.5)	−7.2 (−8.2 to −6.1)	7.0 (5.9 to 8.1)	3.6
	90 to 81 (n = 74)	1.2 (0.4 to 1.9)	−5.4 (−6.8 to −4.1)	7.7 (6.4 to 9.1)	3.5
	≤80 (n = 30)	0.6 (−0.5 to 1.7)	−5.2 (−7.2 to −3.3)	6.4 (4.5 to 8.3)	2.9

^A^—[smartwatch − reference oximeter].

**Table 3:** The sensitivity, specificity, and accuracy of the smartwatches in detecting hypoxemia (SpO_2_ < 90%).

Smartwatch	Sensitivity (95% CI)	Specificity (95% CI)	Accuracy (95% CI)
Apple Watch 8	0.91 (0.85 to 0.97)	0.95 (0.92 to 0.98)	0.93 (0.90 to 0.96)
Samsung Galaxy Watch 5	0.97 (0.94 to 1.00)	0.76 (0.70 to 0.82)	0.84 (0.80 to 0.88)
Withings ScanWatch	0.92 (0.86 to 0.98)	0.86 (0.80 to 0.92)	0.89 (0.85 to 0.93)

## Data Availability

The datasets generated and analyzed during the current study are available in the repository at https://ventilation.fbmi.cvut.cz/data/ (accessed on 8 November 2023).
